# Lymphatic mapping and sentinel node biopsy in gynecological cancers: a critical review of the literature

**DOI:** 10.1186/1477-7819-6-53

**Published:** 2008-05-20

**Authors:** Ali Ayhan, Husnu Celik, Polat Dursun

**Affiliations:** 1 Department of obstetrics and gynecology, division of gynaecological oncology, Baskent University school of medicine, Ankara, Turkey; 2 Department of obstetrics and gynecology, Firat University school of medicine, Elazig, Turkey

## Abstract

Although it does not have a long history of sentinel node evaluation (SLN) in female genital system cancers, there is a growing number of promising study results, despite the presence of some aspects that need to be considered and developed. It has been most commonly used in vulvar and uterine cervivcal cancer in gynecological oncology. According to these studies, almost all of which are prospective, particularly in cases where Technetium-labeled nanocolloid is used, sentinel node detection rate sensitivity and specificity has been reported to be 100%, except for a few cases. In the studies on cervical cancer, sentinel node detection rates have been reported around 80–86%, a little lower than those in vulva cancer, and negative predictive value has been reported about 99%. It is relatively new in endometrial cancer, where its detection rate varies between 50 and 80%. Studies about vulvar melanoma and vaginal cancers are generally case reports. Although it has not been supported with multicenter randomized and controlled studies including larger case series, study results reported by various centers around the world are harmonious and mutually supportive particularly in vulva cancer, and cervix cancer. Even though it does not seem possible to replace the traditional approaches in these two cancers, it is still a serious alternative for the future. We believe that it is important to increase and support the studies that will strengthen the weaknesses of the method, among which there are detection of micrometastases and increasing detection rates, and render it usable in routine clinical practice.

## Background

Sentinel lymph node is the first node where primary tumor lymphatic flow drains first, and therefore the first node where cancer cells metastasize. Lymphatic metastasis has always been a focus of interest for the surgeons, as it is one of the first and foremost routes of spreading in many tumors and, because it shows the level of spreading. The condition of the lymph notes has vital importance in the planning and management of the treatments of many cancers.

Lymphatic mapping is the passage of a marking dye or radioactive substance, injected by a tumoral or peritumoral injection, through the lymphatic vessels draining the primary tumor, that is, afferent lymphatic vessels, to the sentinel lymph node. This lymph node is the one with the highest possibility of involvement in case of metastasis from the primary tumor. According to lymphatic mapping hypothesis, if the sentinel node is negative in terms of metastasis, then non-sentinel nodes are also expected to be negative in that regard. However, there may be metastasis in the non-sentinel nodes even when the sentinel node is negative in terms of metastasis, due to reasons both explicable and inexplicable. Therefore there are reports of false negativity in literature studies [1].

Sentinel lymph node biopsy concept was first developed to identify lymphatic metastasis in parotid carcinoma [2]. Later on, it has been used in penile carcinoma, breast, melanoma, lung, gastrointestinal, endocrine and gynecological cancers. Results of the studies about and experiences in gynecological cancers, particularly vulva cancer, and cervix cancer, as well as endometrial cancer, but to a lesser degree, have been published in the literature. The present study focused on the literature data about the results of the use of sentinel lymph node biopsy concept in gynecological cancers.

## Technique

Several techniques have been reported to identify the sentinel nodes. These are blue dye labeling, radiolabeling and combined labeling that comprise sequential application of blue dye and technetium labeling. Most basically, a vital dye like isosulfan blue is injected into intact tissue that around of tumor intra-operatively. The injections are made in to junction of the tumor and normal tissue in vulvar lesions, peritumoral cervical stroma in cervical cancer circumferentially. In the case of endometrial carcinomas the site of injection are not as well defined. This substance is inert, and rarely causes allergic reactions. Studies report that the highest rate of allergic reactions is 3% [3]. The dye injected reaches the lymph node through microlymphatics in about 5 minutes and the median stain time of dye in the sentinel lymh node is 21 minutes [4].

The second type of mapping is injection of a radiocolloid or both. This procedure requires peritumoral injection tissue that surrounding the tumor of ^99m^TC (Technetium) labeled colloids such as sulfur colloids, albumin colloids or carbon colloids. Although a number of protocol variations have been reported, radiocolloid is injected usually 2–4 h preoperatively if ^99m^Tc sulfur colloid is used and on pre-op day 1 if ^99m^Tc albumin is used. Radiocolloid transported to the sentinel node is identified with a gamma counter applied to the patient. The time interval for maximum tracer accumulation in sentinel node is 1.5 hour after injection [4]. The particle size of labeled colloid is important and the time interval between aplication and detection is affected from particle size. It has not beeen detected any sentinel nodes in the paraaortal region similarly if particles over 200 nm [5].

If the radioisotopes are employed, a preoperative radiolymphoscintigram is performed to detect in localization of the sentinel node(s). Pre-operative lymphoscintigraphy is particularly useful in cases where the primary tumor has more than one drainage. If a preoperative radiolymphoscintigram was performed, this image is used to guide the site and size of the incision and to localize the sentinel node in vulvar cancers. Mostly, dissection of the sentinel node is performed during of surgery in the operation room. The organization of preoperative radiocolloid application and subsequent lymphoscintigraphy is difficult and costly. It has been reported that "Short Tc protocol" without preoperative lymphoscintigraphy has a high detection rates, an easier management and is cost effective [5].

The using of laparoscopic gamma probe is very important alternative in the minimally invasive procedures. After sentinel node is detected and excised gamma counter is used to assess for background radiation that indicates if the correct node has been removed or if there is another sentinel node. The background radiation count should not exceed 10% of the count from the sentinel node. Nodes are usually re-examined with the probe ex vivo to confirm radioactivity, and the lymphadenectomy site is reassessed to exclude residual radioactivity. Sentinel nodes are sent for pathological evaluation as separate specimens [6].

## Vulva cancer

Vulvar carcinoma affects 4% of all gynecological cancers, and is in the fourth most common female genital cancer. Of the cases, 90% are squamous cell carcinomas, while the rest are melanoma, adenocarcinoma, basal cell carcinoma and sarcoma [7].

Nodal metastasis in vulva cancer is the main prognostic factor, irrespective of the size of the primary tumor, and its presence is markedly correlated with survival. Five-year survival was reported 90% in those without inguinal node involvement, 80% in those with two or more nodal involvements, and 12% in those with three or more nodal involvements [6-8]. The risk of involvement is 11% in stage I cases and 25% in stage II cases with stromal invasion over 1 mm. For this reason lymph node dissection should be performed in addition to local excision [6].

Although less radical approaches have been developed with increasing frequency particularly in the last 25 years, postoperative complications still occur at a remarkable rate. Complications like 69% leg edema and 85% injury opening reported in the classical treatment of vulva cancer were reported 19% and 29%, respectively, in a study by GOG, where radicalness was reduced with radical hemivulvectomy and ipsilateral lymphadenectomy in clinical stage I cases [9-12]. However, for the time being, there is not any non-invasive technique that can reliably show nodal metastases. In a metaanalysis carried out by Selman *et al*., sensitivity and specificity of methods used to identify nodal metastasis were reported 72% and 100% in fine needle aspiration, 71% and 72% in positron emission tomography, 86% and 87% in magnetic resonance imaging, 45–100% and 58–96% in ultrasonography [1]. Therefore, non-invasive and/or micro-invasive methods are studied in the hope that they will reduce complications, in addition to exercising a positive effect on survival of patients with vulvar cancer. Of these, the most contemporary and promising method is sentinel lymph node biopsy.

Its applicability has been demonstrated firstly by Levenback *et al*., using isosulfan blue dye on 9 vulvar cancer patients, of whom 7 had squamous cell carcinoma and 2 had melanoma [13]. About a year later, the same authors published a second report on 21 vulvar cancer patients. This study which reported the results of using intra-operative lymphatic mapping with isosulfan blue dye, included 9 T1 cases, 10 T2 cases and one T3 case, as well as one case who had undergone local excision and therefore was not known. Of the lesions in the cases, 10 were lateral and 11 were midline. The study reported a 62% sentinel node detection rate and 100% sensitivity and specificity. It was stated that the cases who had negative sentinel node were not found to have metastasis in non-sentinel nodes. Sentinel nodes were identified in different areas of the superficial compartment [14].

Sentinel node detection rates as low as 60% and rates of failure to detect sentinel node as low as 40%, found in sentinel node studies using isosulfan blue, have caused disappointment at first [1]. DeCesare *et al*., demonstrated the applicability of intra-operative gamma ray use, and a year later, Hullu *et al*., demonstrated the applicability of a combined technique that included pre-operative lymphoscintigraphy and intra-operative blue dye methods [15,16]. It has been reported that avarage detection rate of sentinel nodes in a literature review of vulvar cancers is 85% with blue day only, 99% with radiolabeled (with or without blue day) [17].

At present, quite high identification rates [1] and low false negativity rates are reported in sentinel node procedure employing the combined technique. Puig-Tintore *et al*., reported in a study including 26 patients with vulvar squamous cell carcinoma that sentinel node was detected in 96% of the patients with technetium-99m-labeled (^99m^Tc) and blue dye peritumoral injection. Of these nodes, 76% were unilateral, and 24% were bilateral. It was reported in the concerned study that all non-sentinel nodes were found negative in cases who were not clinically suspected and who had negative sentinel lymph node [18].

In this respect, sentinel lymph node biopsy is a method that needs to be studied and developed, while it must be stressed that large studies are needed to reveal sensitivity, specificity, positive and negative predictive values. However, both the rare incidence of vulva cancer relative to other gynecological cancers and the requirement of a distinct experience for this method limit access to such information. The studies associated with vulvar cancer that included more than 20 cases were presented Table 1.

**Table 1:** Literature review of Sentinel node detection in vulvar cancers (Only Studies with more than 20 patients were presented)

**Author**	**Year**	**Detection method**	**Tracer**	**No. of cases**	**Groins dissected (n)**	**Detection rate (%)**	**Positive SN (n)**	**False negative SN (n)**	**NPV (%)**	**Ultra-staging**
Levenback [14]	1995	BD	-	21	29	66	5	0	100	(-)
De Hullu [16]	1998	ILS+ BD	Nanocolloid	59	107	100	24	0	100	(+)
Ansink [20]	1999	BD	-	51	93	56	9	2	95	(-)
Levenback [19]	2001	BD	-	52	76	88	10	2	100	(+)
Sideri [27]	2000	ILS	Colloid albumin	44	77	100	13	0	100	(-)
De Cicco [28]	2000	ILS	Colloid albumin	37	55	100	8	0	100	(-)
Sliutz [29]	2002	ILS+ BD	Microcolloidal albumin	26	46	100	9	0	100	(+)
Puig-Tintore [18]	2003	ILS + BD	Nanocolloid	26	37	96	8	0	100	(+)
Moore [30]]	2003	ILS + BD	Sulfur colloid	21	31	100	7	0	100	(+)
Hauspy [31]	2007	ILS+ BD	Sulfur colloid	41	68	95	18	0	96	(+)

Abbreviations ; BD: blue dye method, ILS: intraoperative lymphoscintigraphy, NPV: negative predictive value, SN: sentinel node, (+): yes, (-):No,

Although lymphatic mappings appear promising in theory, it has some aspects, which overshadow its success and prevent its liberal use. The first of these aspects is the learning curve. In a sentinel node study carried out using intra-operative isosulfan blue, sentinel nodes were identified in 22 out of 25 patients with a lateral tumor, and 24 out of 27 patients with a midline lesion, consequently in 46 out of a total of 52 patients (88%), False negativity was 0%. The same study failed to identify sentinel nodes in 2 out of 12 groins, which had been proven to have metastatic disease. The authors attributed this to their being in the first two years of the study [12]. The second aspect is false negativity. Although it is reported more commonly in patients in whom blue dye is used, it was also noted in studies where radioactive substance was employed. In two studies with more than 50 cases, Ansink *et al*., reported false negativity in 2 cases in a 51-case series, and Levenback *et al*., reported 2 in a 52-case series respectively [19,20].

The third and maybe the most current aspect is the case of patients who are found negative in terms of metastasis on histopathological evaluation, but are identified by ultrastaging to have metastasis at the micro level. In the study by Puig-Tintore *et al*., rate of micrometastasis identifiable by ultrastaging was established as high as 38%. The concerned study which included squamous cell vulvar carcinoma patients found sentinel lymph nodes in 96% of the cases with ^m ^and blue dye peritumoral injection. Of these nodes, 76% were unilateral, and 24% were bilateral. In the study, all the non-sentinel lymph nodes were found negative in cases who were not clinically suspected and whose sentinel lymph nodes were negative. Negative predictive value was reported 100% [18]. When the pathologically negative sentinel nodes were subjected to microstaging with serial sections, and immunochemically stained with cytokeratin, micrometastasis was found in 11% of sentinel nodes, which were negative by hematoxylin eosin stain [21]. In a study by Terada, sentinel lymph nodes were made in 10 cases, and sentinel nodes were obtained in all. One node was found positive and the others negative by conventional staining. Serial sectioning and immunohistologic staining showed two metastases in these cases. Two out of the three positive nodes could not be identified by conventional histopathological evaluation [22].

Recurrence was reported 6% in cases in whom sentinel lymph node biopsy was conducted. Of the 52 cases included a sentinel lymph node study by Frumovitz *et al*., those who had recurrence were reported in a study. It was noted in the concerned study that of the cases in whom lymphatic mapping was conducted, recurrence developed in three cases with squamous vulvar cancer. A retrospective investigation revealed that one of these cases had positive SLN, positive non-SLN and extracapsular disease, and was at high risk for recurrence, the other was a case in whom sentinel node was not identified, and the third was a case who had negative sentinel node and negative non-sentinel node. It was reported that the last case was identified to have bilateral sentinel node in the clitoral lesion, and was negative in the conventional histological evaluation [23].

In conclusion, sentinel lymph node concept that was developed to avoid severe complications like injury infections, injury opening and lymphedema caused by inguinofemoral lymphadenectomy performed in addition to radical vulvectomy in vulvar cancer, which is seen rarely relative to other gynecological cancers, but is an extremely destructive disease, is a promising method in terms of its applicability in routine clinical practice. Micrometastasis, which overshadows the success of the method, appears like a problem that can be overcome by ultrastaging and immunohistochemistry. A study comparing complete inguinofemoral lymph node dissection and sentinel node procedure results did not show any difference between the rates of metastatic lymph nodes excised by two methods, whereas identification of micrometastases was found higher by sentinel node biopsy and ultrastaging, than by complete inguinofemoral lymph node dissection [24].

An extensive phase III study, exploring the negative predictive value of a negative sentinel lymph node in stage I and II invasive squamous cell vulvar cancers and the localization of the sentinel node in these patients, is still under way in the National Cancer Institute (GOG-173).

## Vulvar melanoma

This is the second most common vulvar cancer after squamous cell cancer. The only effective treatment among available treatments is surgery, and the role of elective lymphadenectomy is debatable. Thus, there is only limited experience with sentinel lymph node. One of the major studies in the literature is the one conducted by De Hullu *et al*., [25]. In the concerned study, complete inguinofemoral lymph node dissection was performed in three cases, who had positive sentinel node, out of 9 vulvar melanoma cases. All of the dissected sentinel nodes were found negative in terms of metastasis in routine histopathologic examination in these cases, except for one, in whom additional nodal metastasis was detected. Immunohistochemical investigations of these nodes conducted by step-sectioning and S-100 and HMB-45 were also found negative. Follow-up of the cases who underwent sentinel node procedure showed recurrence in two patients. Authors of the study recommended the use of sentinel lymph node procedure only within the context of clinical studies. In another study, Abramova *et al*., described experiences with lymphatic mapping and the following sentinel node biopsy procedure using ^99m^Tc -labeled sulfur colloid in 6 patients with vulvar melanoma. These researchers who also collected the cases in the literature reported that the success in identifying the localization of the sentinel node was about 100% [26]. Other series on vulver cancer are drtailed in table 1[27-30]

## Cervical cancer

Pelvic nodal involvement in early stage cervical cancers eligible for surgery was reported 0–4.8% in Stage IA, 17% in Stage IB, 12–27% in IIA and 25–30% in IIB [31,32]. Basically, systemic retroperitoneal lymph dissection is performed in all these cases to identify nodal involvement, which is seen at a rate of 0–4.8% in Stage IA. This means that the performed lymphadenectomy procedure will not benefit more than 90% of cases, and besides, these patients can face such complications as prolonged operation time, blood loss, blood transfusion, lymphocyst, and lymphedema. Therefore, sentinel lymph node procedure aimed to reveal the nodal condition has been an increasingly popular topic of research in cervix cancer on the same grounds with vulvar cancer. It has been presented literature review of sentinel node detection in cervical cancer in table 2.

**Table 2:** Literature review of sentinel node detection in cervical cancers (Only Studies with more than 20 patients were presented)

**Author**	**Yıl**	**Detection method**	**Tracer**	**Surgery**	**No. of cases**	**Lymph node dissection**	**Detection rate (%)**	**Positive SN**	**False negative SN**	**NPV (%)**	**Ultrastaging**
Malur [44]	2001	ILS or BD	Albumin-RES	LT/LS	50	PN+PAN	80	6	1	97	(-)
Rhim [34]	2002	ILS + BD	Colloid albumin	LT	26	PN+PAN	100	5	1	95	(-)
Levenback [36]	2002	ILS + BD	Radiocolloid	LT	39	PN+PAN	100	8	1	97	(+)
Plante [2]	2003	BD	Antimony trisulfide colloid	LS	41	PN+PAN	79	12	0	100	(+)
Martinez-Palones [37]	2004	ILS + BD	Colloid albumin	LT/LS	25	PN+PAN	92	4	0	100	(+)
Chung [48]	2003	ILS + BD	Sulphur colloid	LT	26	PN+PAN(bifurcation)	100	1	0	100	?
Buist [49]	2003	ILS + BD	Colloid albumin	LS	25	PN	100	9	1	94	(+)
Hubalewska [50]	2003	ILS + BD	Nanocolloid	LT	37	PN+PAN	100	5	?	?	?
Van Dam [51]	2003	ILS	Nanocolloid	LS	25	PN	84	5	0	100	?
Marchiole [53]	2004	BD	-	LS	29	PN	100	2	3	87.5	(+)
Niikura [54]	2004	ILS + BD	Phytate	LT	20	PN	90	2	0	100	(+)
Pijpers [55]	2004	ILS + BD	Colloid albumin	LS	34	PN	97	17	1	92	?
Silva [42]	2005	ILS	Phytate	LT	56	PN	93	10	3	92	(+)
Rob [5]	2005	BD	-	LT/LS	100	PN+PAN	80	20	1	99	(+)
Di Stefano [56]	2005	BD	-	LT	50	PN	90	9	1	97	(+)
Angioli [57]	2005	ILS, (LS+BD)	Colloid albumin	LS	37 (83)	PN	70 (96.4)	9 (15)	0 (0)	100(100)	(+)
Lin [58]	2005	ILS	Sulfur colloid	LT	30	PN	100	7	0	100	(+)

BD: blue dye method, ILS: intraoperative lymphoscintigraphy, LS: laparoscopy, LT: laparotomy; NPV: negative predictive value, SN: sentinel node, (+): yes, (-):No, ?: Unknown, PN: Pelvic lymph node dissection, PAN: Para-aortic lymph node dissection

Sentinel lymph node biopsy, which is less invasive and cheaper, and has a lower rate of morbidity. However, some serious restrictions need to be clarified for the method to be applicable in clinical practice. The main restrictions include distribution of sentinel lymph nodes over a wider area due to the lymphatic distribution of the cervix, localization of the tumor in the cervix, and a resulting lower detection rate, and sensitivity, as well as higher false negativity. These conditions are complementary to the technique and are used to evaluate the dissected lymph nodes.

The known lymphatic distribution of the cervix has three different lymphatic patways have been identified; laterally to external iliac and common iliac nods, internally to the hypogastric nodes, and posteriorly to the pre-sacral and then para-aortic nodes. Although majority of the nodes are located in internal iliac and external iliac areas, nodes have been found in also presacral, parametrial and pararectal areas [33]. In a sentinel node study carried out with 26 patients using combined technique, Rhim CC *et al*., found that of the sentinel nods 18 were in the external iliac, 12 in the obturatory, 8 in the internal iliac, 8 in the parametrial, 2 in the common iliac and one in the inguinal lymph nodes [34]. In a study by O'Boyle et. al. 17% of the sentinel nodes were found in the common iliac area, 62% in the external iliac, 4% in the internal iliac, and 17% in the parametrial areas [35], whereas Levenback found 9% of the sentinel nodes in the paraaortic area, 11% in the common iliac, 71% in the external iliac, and 9% in the parametrial area in a study including stage IA-IIA cases [36]. Martinez Palones found in his study with 26 cases that of the sentinel nodes, 40% were in the internal iliac and 25% were in the external iliac area [37]. Barranger obtained 67% of the sentinel lymph nodes in the external iliac area, 28% in the internal iliac area, and 5% in the common iliac area [38]. Although different studies report different results, sentinel lymph nodes are most commonly identified in the external iliac area, which is followed by common iliac and parametrial areas, in most of the studies. These localizations are consistent with the results obtained by conventional complete lymphadenectomy [38-41]. In their study Rhim *et al*., reported that of the 21 cases whose sentinel lymph nodes were found negative, pelvic lymph nodes were also negative in all, but one case. Of the 5 cases whose sentinel lymph nodes were positive, 4 were found to have pelvic lymph nodes positive, and one negative. In this study sentinel node detection rate was reported 94%, overall accuracy 97%, and false negativity 4.76% [34].

Presence of micrometastases has been reported in sentinel node studies including cervical cancer cases as well. In the lymphatic mapping study conducted by Silva *et al*., using ^99m^Tc labeled phytate, it was reported that micrometastases were established by cytokeratin immunohistochemical in 5.1% of the sentinel lymph nodes which were negative by hematoxylin eosin. In the concerned study, 44% of the sentinel nodes were found in the external iliac, 39% in the obturatory, 8.3% in the internal iliac and 6.7% in the common iliac area, and sensitivity was reported 82.3%, NPD 92.1%, and accuracy of sentinel node in predicting lymph node condition 94.2% [42]. In the study by Levenback, sentinel node sensitivity was found 87.5%, negative predictive value 97%, and false negativity 11% [36], while Ying *et al*., established in their study that the detection rate of sentinel lymph node biopsy was 75%, and sensitivity, specificity and accuracy were 75%, 100% and 95%, respectively [43].

Not only the amount of blue dye used in sentinel node studies in cervical cancer affects the rate of identified sentinel nodes, but also use of radioactive isotope instead of dye as a marker influences the sentinel node detection rate. In a study where they conducted sentinel node research with Patent Blue Violet in all cases before systemic lymph node dissection, Dargent *et al*., investigated the changes in sentinel node detection rate in proportion to the amount of dye used. They reported that when they used 1.5 ml of dye or less, they found 50% of the sentinel nodes, and when they used 4 ml of dye, they found 90%of the sentinel nodes [39]. Malur *et al*. studied sentinel node detection rate, sensitivity and negative predictive value using radioactive isotope instead of dye only, and a combination of the two [44]. Sentinel node detection rate in this study was 55% with blue dye only, 76% with radiolabeled and 90% with the combined technique. Sensitivity and negative predictive value, which were 83.3% and 97.1% respectively, reached 100% when dye and radioactive isotope were used in combination. Similarly, false negativity rate, which was 16% dropped to 0%. In a study by Plante *et al*., the detection rate which was 79% by dye alone rose to 93% with the addition of lymphoscintigraphy. Negative predictive value of the combined technique was reported at 100% [3]. Likewise, in a study by Lambaudie et. al., sensitivity was 33%, specificity 100%, PPD 100%, and NPD 100% when dye was used alone, as opposed to dye and isotope combination where sensitivity was 66%, specificity 100%, PPD 100%, and NPD 90% [45].

Use of laparoscopy with a view to making the procedure less invasive has also been investigated in sentinel node biopsy studies. In this context Barringer *et al*., conducted a sentinel node study using radioactive isotope and patent blue combination with the help of an endoscopic gamma probe before complete laparoscopic pelvic lymphadenectomy in 13 patients. Twelve out of 13 patients were found to have sentinel lymph nodes (92%). One patient was found to have only one microscopic metastasis by immunohistochemical examination [38]. In short, detection rate, sensitivity, specificity, and negative predictive value are reported to increase, while false negativity decreases in studies where lymphoscintigraphy is added to blue dye use. Allergic reaction at a rate of 3% and the longer learning curve reported in dye use indicate that radioisotope is more advantageous in cervical cancer [3]. Previous conization and stage is not necessarily a cause of failure. Effect of diagnostic conization, on the sentinel node detection rate is controversial. I has been reported no advers effect in most of studies associated with previous conization [36,39,45], whereas in a study lower detection rate has been founded [46].

Addition of such modalities as radioisotope use and laparoscopy use to sentinel node studies in cervical cancer helps to increase the success of the method. In order to further develop the method, progress should be achieved in increasing the accuracy of frozen examinations in sentinel node procedure, as whether or not to continue to lymphadenectomy should be decided on the basis of the information pertaining to the sentinel node. Sensitivity and specificity of the sentinel node frozen biopsy are currently reported 95.2% (20/21) and 80% (4/5) in cervical cancers respectively [34]. However, it may be difficult to identify metastases by sentinel node frozen biopsy. Multiple cross sections of the dissected node and immunohistochemical staining may help compensate for this false negativity, although these methods are time-consuming and do not seem practical for the purposes of frozen biopsy.

Determining sensitinel node using preoperative SPECT/CT lymphoscintigraphy is the newest progress in sentitinel node of cervix cancer. This Technique is very similar to conventional nuclear medicine planar imaging using a gamma camera. However, it is able to provide true 3D information. Kushner et al studied in 20 cases and they found sensitinel node: 33% as obturauar, 30% as external iliac, 19% as internal iliac area. Interestingly sensitinel node were found in unusual area, e.g.11% as common iliac, 5% as presacral, 3% paraaortic. In this study, lymphoscintigraphy detection rate was reported as 100% NPD [47]. In conclusion, in order for sentinel node biopsy to replace conventional approaches with its practicality and reliability, prospective studies with larger case series are needed in cervical cancers. Other studies are detailed in table 2[48-58].

## Endometrial cancer

Endometrial cancer is the most common gynecological cancer in industrialized countries. Involvement of pelvic and paraaortic lymph nodes is a very important prognostic parameter in endometrial cancers. Upstage resulting from nodal involvement was found in 12.4% of clinical stage I cases, and 27.3% of clinical stage II cases [59]. Therefore, the stage should be exactly determined in order to obtain information about the prognosis of the patient and to plan adjuvant treatments. Lymphadenectomy procedure is the standard in staging surgery to reveal the condition of the lymph nodes. As in other gynecological cancers, increase in morbidity and complications associated with lymphadenectomy have led to research about the less invasive sentinel node concept in endometrium cancer.

Since the lymphatic network of the uterus is more complex than that of the cervix and vulva, and it is more difficult to access the dye or radioisotope injection area, sentinel lymph node studies in endometrial cancers are rarer, relative to those in other cancers. In a study where sentinel node examination was conducted in 15 high-risk endometrial tumor cases using subserosal isosulfan blue dye injection during laparotomy, 10 cases (67%) were found to have dyed lymph nodes, and of a total of 31 lymph nodes dissected from these cases, 12 were reported to be in the paraaortic area, 6 in the common iliac area, and 13 in the pelvic region. [60]. In a lymphatic mapping study where patent blue-V was injected into the uterine wall by laparoscopy, instead of laparotomy, in 8 cases, 5 cases (62.5%) were found to have sentinel nodes in the obturatory, internal iliac and common iliac areas [61].

In their study where they explored the changes in sentinel node detection rate by the injection site of patent blue-V dye, Holub et al., injected patent blue-V dye into the subserosal myometrium in 13 out of 25 patients, and into the cervico-subserosal myometrium in 12 patients. Sentinel node detection rate was 61.5% in the subserosal myometrium group, and 83.3% in the cervico-subserosal myometrium group. Although there was not any statistical difference between the groups, it was reported that the mean number of sentinel nodes identified per case was significantly higher in the cervico-subserosal myometrium group [62]. In another study, by Gien *et al*., isosulfan blue dye injection was made by hysterescopy during laparotomy into the peritumoral endomyometrium, subserosa, or both in 16 cases. Sentinel nodes were identified in 56% of the cases to whom only serosal injection was made, and 50% of those in whom both serosal and hysterescopic injection were made. Overall sentinel node detection rate was found 44%, and negative predictive value, 86% [63].

Microscopic metastasis has been explored in sentinel node studies with endometrium cancer. In a laparoscopic sentinel node study where a total of 11 cases, of whom 10 were stage IB and one stage IIA, were injected with re-operative radioactive isotope and intra-operative blue dye, Pelosi *et al*., found metastases in three out of 17 lymph nodes (17.5–6%), of which 6 were bilateral and 5 were unilateral [64]. Again, Pelosi *et al*., investigated the prognostic role of sentinel lymph node biopsy procedure in a study where sentinel nodes, all of which were in the internal iliac lymph nodes of 15 out of 16 patients (93.7%) were identified by lymphoscintigraphy and laparoscopically-assisted intra-operative sentinel lymph node biopsy in 16 patients with FIGO IB endometrial cancer. They found micrometastases in 3 out of the 24 lymph nodes, and reported that there was no relapse in the 12 cases whom they could follow-up [65]. In another study where sentinel node was explored by pre-operative lymphoscintigraphy and intra-operative gamma probe, sentinel nodes were identified in 82% of the 28 endometrial cancer cases. The tumor in 95% of the cases in whom sentinel nodes were identified was found to have 50% invasion. These researchers attributed the high identification rates to the sentinel node modality and hysterescopy they used [66].

In a prospective study where they examined sentinel lymph nodes by hysterescopic pre-operative peritumoral m Nanocolloid injection and lymphoscintigraphy, Fersis *et al*., reported 85.7% sensitivity [67]. In another sentinel node study that used the combined technique, hysterescopic subendometrial peritumoral m -Nanocolloid and blue dye injection in 26 cases, sensitivity was reported 100% [68]. The fact that involved lymph nodes in the endometrium are examined over a wide retroperitoneal area in cases where blue dye is used brings about a serious decrease in the detection rate due to the dye's rapidly passing through the lymphatics. Although pre-operative lymphoscintigraphy seems more sensitive than blue dye, it has been argued that intra-operative follow-up with a gamma probe is even more sensitive. In a study by Nikura *et al*., sentinel nodes that could not be identified by pre-operative scintigraphy in 4 cases were identified intra-operatively [66].

An interesting case reported by Van Dam *et al*., has added a different dimension to the sentinel node concept. A case of stage IB, grade 2 endometrial cancer, who was treated with total abdominal hysterectomy, bilateral salpingoopherectomy, pelvic node sampling and vaginal vault radiation, and developed mid-vaginal recurrence after the treatment, was studied in terms of selective lymph node by peritumoral technetium nanocolloid injection, and was found to have a total of 3 sentinel nodes, two in the left obturatory fossa and one in the right external iliac region. When these were found normal on histology, total vaginectomy, parametrectomy and pelvic lymphadenectomy were performed [69]. In conclusion, although results of studies about sentinel node research in endometrial cancer are promising, though not to the same extent with those in vulvar and cervical cancers, further studies are needed.

## Vaginal cancer

Number of literature studies about sentinel node procedure in vaginal cancer patients is fairly scarce. Of these, the main study is the one where Vam Dam *et al*., reported 4 cases. In the concerned study, sentinel node procedure was performed in primary and recurrence vaginal cancer cases. In all cases, pre-operative 60-mBq technetium-labeled nanocolloid injections were made at 3, 6, 9, 12 hour lines, adjacent to the cancer in the vagina, which was followed by dissection of sentinel nodes laparoscopically or with a handheld probe. Sentinel nodes could be identified in two out of the three patients. Sentinel nodes were found in the groin and obturatory area in one case, and just below the junction of iliac vessels in the other. Sentinel node could not be identified by lymphoscintigraphy in one patient. Sentinel node procedure could not be conducted in one patient who was treated with combined chemo-radiotherapy initially, but showed recurrence 6 months later. In this patient, a sentinel node was identified in the right obturatory area during staging procedure, and was dissected laparoscopically. Localizations of the sentinel nodes identified in this study, which were external iliac region and groin in distal vaginal cancers, and obturatory fossa and external iliac region in proximal vaginal cancers, are consistent with our previous knowledge [70].

## Paradoxial conditions in sentinel node biopsy

Although according to sentinel node hypothesis the metastasis in the first node draining the tumor is identified, this is not always the case. There are many cases which cause sentinel node procedure to give false negative results, or where sentinel node cannot be identified. It was reported in a study including vulvar cancer cases that the metastatic lymph node identified by palpation intra-operatively could be bypassed due to lymphatic stasis caused by hardening associated with metastasis, or that sentinel node could not be identified due the stasis of the lymphatic flow [71,72]. Pre-operative and post-operative palpation is important in sentinel node examination due to such findings. Similarly, pre-operative computerized tomography and magnetic resonance imaging can be considered, or nodal biopsy in the accompaniment of USG can be carried out in cases with enlarged lymph nodes. In a study including cervical cancer patients Plante *et al*., found that the rate of sentinel node detection in the dissection area of the patients who had nodes that appeared normal on laparoscopy was 75% and sentinel node detection rate in patients with macroscopic involvement was 75% [3]. Similarly it was noted that sentinel node detection rate decreased in endometrial cancers, where sentinel node experience is lower relative to other gynecological cancers, due to an impairment of the lymphatic flow when myometrial invasion is above 50% [66]. Another finding is that the histopathological examination of a sentinel mass formed by two lymph nodes revealed by lymphoscintigraphy showed that one the concerned nodes was sentinel and the other was non-sentinel. Complete sentinel node dissection will be appropriate in such cases. Besides, the pathologists who conduct the frozen examination should be informed about the number of dissected sentinel nodes. In addition, increased Body Mass Index has a reductive effect on sentinel node detection. Pre-operative USG and directed biopsy can be utilized to decrease these negative results [71].

## Why are micrometastates important and how should the future be?

According to sentinel node concept, negativity of the sentinel lymph node requires other nodes to be negative in terms of metastasis. However, microscopic metastasis in the sentinel node might be interpreted as negative, when evaluated by classical hematoxylin eosin. This is an important condition, and there may be metastasis in non-sentinel nodes in case that there is microscopic metastasis in the sentinel nodes. Indeed, there is no special definitions associated with micrometastases, macrometastases or submicrometastases in gnecologycal cancers and use accepted criterions in breast cancers. According to the Philadelphia Consensus Conference about sentinel node in breast cancer; Any cluster of malignant epithelial cells less than 2 mm in size was designated as mikrometastasis. Inside this category of metastases, any cohesive cluster of malignant cells that 200 μm or less in size was designated as submicrometastases [73]. **This **is very important clinically. Likewise, in a study by Robinson, a metastasis smaller than 2 mm was found in the inguinal node, and metastasis was identified in another inguinal lymph node in this case [24]. Besides, it has been shown in many studies that micrometastasis poses an increased risk in terms of recurrence. In their study including cervical cancers, Juretzka *et al*., reported that recurrence developed in 50% of patients with micrometastasis, and 6.7% of those without micrometastasis [74]. Similarly, relative risk of recurrence was reported 2.44 in early stage cervix cancers, which do not have nodal metastasis in the histopathological evaluation, but do have nodal micrometastasis, and 2.22 in the presence of submicrometastasis [75]. In another study, it was reported that recurrence risk in vulva cancers, where there was not nodal involvement histologically, but presence of metastasis was shown, increased 20 folds relative to the risk in those who do not have micrometastasis [76]. It has been reported that prognostic value of micrometastasis is controversial in some studies [3]

Given the starting point of sentinel lymph node concept, microscopic metastases that dwarf the applicability of the method become more important. This condition which causes false negativity is still pertinent to many tumors. Re-addressing of this condition within lymphatic mapping concept can lend credibility to the method's applicability. It has been argued that the issue can be resolved by the addition of a histopathological ultrastaging protocol to the sentinel node procedure. In Terada's study, 2 out of 14 cases found negative by conventional staining were found positive by ultrastaging, where cross sections are prepared thinner [22]. Van Deist et. al. suggested preparation of additional cross sections with 250 μ intervals and immunohistochemical staining with cytokeratin [77]. However, these methods are time-consuming, and should be balanced with output. Nevertheless, it is also possible to find occult lymph node metastases in 23% of the patients, when the lymph nodes found negative by hematoxylin eosin are stained with cytokeratin AE1/AE3 and serial sectioning [78]. The fact that immunohistochemical staining increases the identification of metastases has also been demonstrated in other studies. In their study Lentz *et al*., found micrometastases at a rate of 15% in the immunohistochemical examination using antibodies against cytokeratins AE-1 and CAM 5.2 in early stage cervical cancer with negative nodes [79]. Of the patients with micrometastases, 75% had lymph-vascular space invasion. Therefore, it was argued that immunohistochemical examination of pelvic nodes could ensure better identification of micrometastases in cases with positive lymph-vascular space invasion [74].

Marchiolè *et al*. proposed an algorithm based on literature data and results of their studies. According to this algorithm, adjuvant therapy is not recommended in early stage cervix tumors which do not have nodal involvement and lymph-vascular invasion, whereas micrometastasis should be examined, and if present, adjuvant treatment should be considered in cases who do not have nodal involvement, but have lymph-vascular space invasion [75].

There are also studies reporting that ultrastaging, the most contemporary and common method recommended for the identification of micrometastases, and immunohistochemical staining do not increase the identification of micrometastasis relative to hematoxylin eosin staining [30]. It is necessary to search new methods that can be applied to clinical practice due to such results, though rare, about the clinical value of additional histopathological techniques and the inadequate output of available methods. Of these, the most current ones are flow cytometry and PCR analyses. Reverse-transcriptase PCR appears to be the most sensitive method to detect metastases. In a study using reverse-transcriptase PCR, Van Trappen *et al*., found occult micrometastases in 50% of early stage cervical cancers [80].

Marchiolè et. al. found micrometastases in 5 cases (19%) with multilevel sectioning followed by cytokeratin immunohistochemistry examinations of the sentinel and non-sentinel nodes of 26 cases with negative nodes, out of 29 early cervical carcinoma cases in whom laparoscopic lymphatic mapping and sentinel lymph node biopsy was performed with patent blue. Of these micrometastases, 2 were identified in the sentinel nodes, and the rest in non-sentinel nodes. Another highly important finding was that the cases who had microscopic metastasis in non-sentinel nodes did not have sentinel node involvement. NPD was 87.5% in this study. Results of the concerned study require a serious questioning of the sentinel node concept [53].

## Sentinel node biobsy and the future

In consideration of the tendency of study results in the literature and contemporary medical approach concept towards non-invasive or at least minimally invasive strategies [81,82], sentinel node procedure, which is minimally invasive, reduces radicalness, and individualizes the patient and the treatment, appears to be a method that needs to be concentrated on, and developed as an alternative to systemic lymphadenectomy, which is considered a major surgery. Conditions that should be met to ensure the successful applicability of sentinel node biopsy concept in gynecological cancers and its replacement of conventional methods in the long-term include increasing experiments related to the method, and presentation of more results from randomized studies. It is necessary to establish standards in the field of histopathological examination, develop frozen examinations, and incorporate nuclear physics departments into the field in order to identify micrometastases. In this point, it should be determined optimal particle size of radioactive tracers and tecniqes of preparation in gynecological cancers.

Learning curve is pivotal. This requires including not only gynecologist oncologists, but also histopathologists and nuclear physics experts into the subject. All these units in the centers where lymphatic mapping is performed should have a sufficient level of knowledge about the concept.

## Conclusion

It has been reported extremely interesting results regarding sentinel node cancer and lymphatic mapping procedure in gynecological cancer. We believe that these results could promise for future gynecological cancer approach. However, there are further study requirements in pathological, nuclear medicine and gynecological oncology areas with regarding sentinel node cancer and lymphatic mapping procedure. This approach has not been in routine use in clinical medicine. Thus, it is important to share with patients to the knowledge of advantage and/or disadvantage obtained from gynecological cancer cases.

## Competing interests

The authors declare that they have no competing interests.

## Authors' contributions

**AA** literature search and drafting of the article

**HC **concept, literature search and helped in drafting

**PD** concept and design, editing of the article

All authors read and approved the final manuscript for publiction.
